# Elucidation of the 5’ End of the Zebrafish *col2a1b* gene at the Telomeric-End of Chromosome 11

**DOI:** 10.17912/micropub.biology.001729

**Published:** 2025-09-09

**Authors:** Angelina R. Carcione, Rodney M. Dale

**Affiliations:** 1 Bioinformatics Program, Loyola University Chicago, Chicago, Illinois, United States; 2 Department of Biology, Loyola University Chicago, Chicago, Illinois, United States

## Abstract

Type II collagens (Col2) play a vital role in the formation of many vertebrate structures, such as the skeleton, the notochord, and many other tissues. This study sets out to clarify the start of the genomic annotation around one of the zebrafish orthologs of vertebrate Type II collagen, *col2a1b ,* as it sits at the telomeric end of chromosome 11 and up till this point has missing coding and non-coding exons form the published databases. We have used bioinformatic tools to elucidate this missing genomic sequence and analyze for basal promoter elements that designate the true first exon of *col2a1b* .

**Figure 1. Elucidation of missing exons and transcriptional start site for zebrafish col2a1b .**
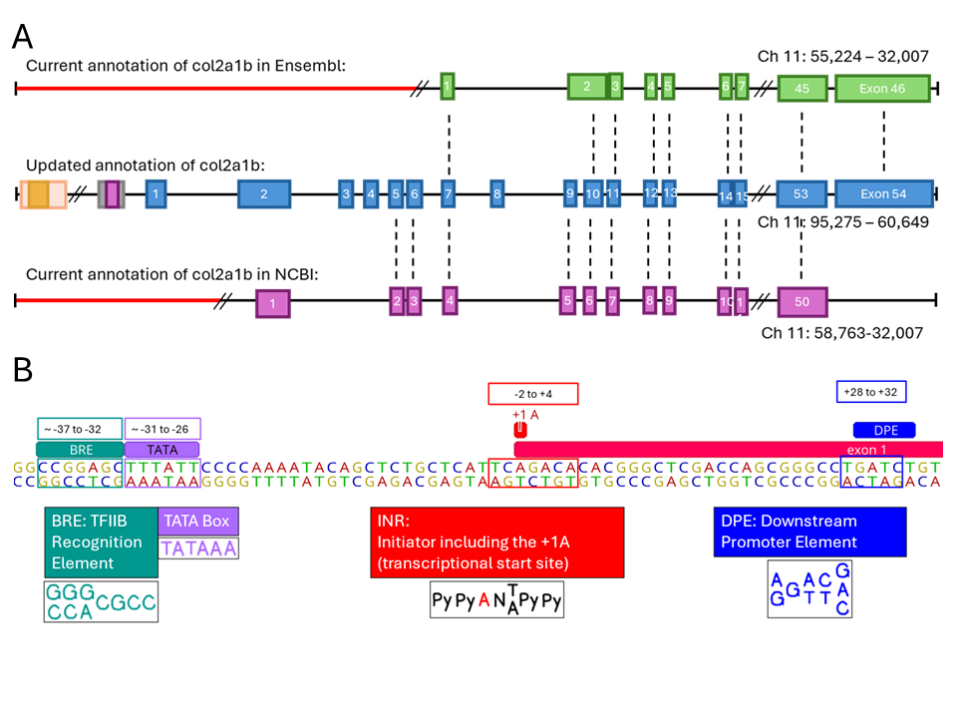
**A.** Schematic of comparisons of the Ensembl (top) and NCBI (bottom) genomic sequence of the *col2a1b* gene compared to our updated sequence annotation (middle). The green, blue, and pink squares represent the exons for Ensembl's annotation, current annotation, and NCBI's annotation respectively. Diagram shows that for both the Ensembl and the NCBI annotations, they are missing exons 1, 2, 3, 4, and 8 in both. Ensembl is also missing exons 5, 6, and 9. NCBI is missing the last exon, updated exon 54, that Ensembl does have labeled as the 46th exon. Ensembl has 46 exons for *col2a1b* in total spanning from 55,224 to 32,007 on the reverse strand of chromosome 11 of the current reference genome: GRCz11 ( GCA_000002035.4 ). NCBI has a total of 50 exons for their annotation spanning from 58,763 to 32,007 on the reverse strand of chromosome 11 of the current reference genome: GRCz11 ( GCA_000002035.4 ) Current annotation of *col2a1b* has 54 exons and spans from 95,275 to 60,649 on Danio rerio strain Tubingen chromosome 11 genome assembly from NHGRI (GenBank: CP137024.3). Orange and yellow square and grey and pink square represent identified regulatory elements and transcriptional promoter elements of col2a1b . Red lines in schematic for NCBI and Ensembl represent the start of poor sequencing quality upstream of the identified first exon. **B** . Schematic of the basal promoter elements for *col2a1b* . BRE (TFIIB Recognition Element), INR (Initiator Element), and DPE (Downstream Promoter Element) were identified.

## Description

In genome browsers Ensembl (Wellcome Sanger Institute) (Dyer et al., 2025) and Genome (National Center for Biotechnology Information), the *col2a1b* gene is marked as incomplete. The most current version of the *col2a1b* gene in GRCz11, Ensembl Release 114 (May 2017), located at the telomeric-end of the zebrafish chromosome 11, has an exon count ranging from 48-52 exons with its length varying depending on the database. Due to its location on the telomere of chromosome 11, its surrounding genome consists of frequent repetitive sequences, making it one of the last regions in the zebrafish genome data to be finalized. The sequencing of telomeric ends is difficult due to issues with DNA polymerases and assembling genome sequences in repeat areas requires sequence reads longer than the length of the repeated sequence; resulting in assemblers not knowing how long the repeat is and where to place sequences in between the repeats. The telomeres are comprised of dozens to thousands of repeats placed by telomerase typically resembling a GT-rich 5’TTAGGG-3’ (Hatakeyama et al., 2013). Thanks to modern sequencing technologies, repeats are more easily sequenced.

To identify the upstream region of our gene, we used data from a previous project done in our laboratory that involved sequencing the 5’ region of the *col2a1b* gene using PCR genome walking. This data was an annotated sequence file with 5,000 bases including the UTR of the first exon of *col2a1b.* Utilizing this data we searched for more recent sequencing data in the public databases. Using the National Center for Biotechnology Information (NCBI)’s Basic Local Alignment Search Tool (BLAST), we found a more recent chromosome 11 sequencing done at National Human Genome Research Institute (NHGRI) from 2023, CP137024.2, which was used for this study. There has been a more current version of the chromosome released in March 2025, CP137024.3, after the completion of this study. This updated version does not change the *col2a1b* gene data we found besides the location of the gene on the chromosome, now from 95,275 to 60,649 base pairs on the reverse strand. Exons were annotated by mapping verified *col2a1b* mRNA. The mRNA mapped with 100% identity, outside the poly A tail. This elucidated the true 5’ untranslated and coding regions of the *col2a1b* gene, as well as its true length. As can be seen in Figure 1, The *col2a1b* gene now has the full 54 exons and 34,627 base pairs, updating 9 exons to Ensembl, and 4 exons to NCBI databases.

With our updated annotation of the *col2a1b* gene, we identified the transcriptional start site, as well as the transcriptional minimal promoter sites BRE (TFIIB Recognition Element), INR (Initiator Element), and DPE (Downstream Promoter Element) (Figure 2). These minimal promoter elements are typically found in the same range from the +1A site we have identified. Finding these binding sites verifies that we had found the transcriptional start site and the start of *col2a1b* .

## Methods

Correction and Completion of col2a1b Gene Sequence in silico

1.1: Identification of col2a1b Sequence and Exons

In Ensembl, GenBank, Zfin, and UCSC genome browser, all current versions of zebrafish *col2a1b* sequence are incomplete. The number of exons, as well as the length of the genes, are inconsistent across all databases. The Ensembl Release 114 (May 2017) ,GRCz11, assembly was submitted May 9, 2017, assembled using clone sequences, and whole genome shotgun sequencing. Overall, this is the most current genome sequence for zebrafish, but it still has many gaps and sequencing errors. To reconcile these issues, past work in our laboratory included PCR genome walking to discover the telomeric region of chromosome 11 which should include all *col2a1b* . Using the 5,000 base pair region resulting from this PCR genome walking, we identified an unpublished “Telomere-to-telomere assembly of Danio rerio Tubingen double heterozygous isolate 11” which is the pseudomolecule sequence for chromosome 11, CP1327024.2. BLAST revealed that the section of the genome we had discovered was not in the current GRCz11 genome assembly. This submission was submitted to NCBI in November 2023 and as part of the recent whole genome sequencing done by the National Human Genome Research Institute (NHGRI). More recently a new assembly has been added to NCBI since completion of our work, GCF_049306965.1, GRCz12tu (April 2025). This new assembly correlated with our analysis and still allowed us to identify col2a1b’s location and provided sequence that was used to identify promoter elements, exons, and regulatory elements.

1.2: 5’ col2a1b Gene Annotation

To discover the conserved regions of col2a1b, the 5’ end of the gene needed to be properly annotated to find the transcriptional start site, promoter elements, and conserved regulatory elements. Prior to this annotation, the downstream exons had been identified, however, due to the proximity of the 5’ end to the gene to the end of the telomere, the current Ensembl genome assembly, GRCz11 had gaps in our region of interest, so the exons could not be identified. Determination of the exons was conducted through alignment of the mRNA (NM_001281478.1) to our newly finished genomic DNA (CP1327024.2.). The alignment was done using the genome assembly program Geneious. In Geneious, we used the “align/assemble” tool and then used “map to reference” to map the mRNA, NM_001281478.1, to our reference, CP1327024.2. The resulting alignment showed where in the gene sequence the exons would be located. From there, exons were annotated and extracted to make sure that the mRNA had been fully captured. The annotation was done manually, while visualization and alignment was completed using bioinformatic software Geneious Prime 2025.0.

1.3: Identification of col2a1b Promoter Regions

Once the gene had been properly annotated, identification of the transcriptional start site, initiator element (INR), downstream promoter element (DPE), TATA box (TATA), and TFIIB recognition element (BRE) were discovered to verify that the 5’ end of the gene had been properly identified. We manually identified these core promoter elements as they help position RNA polymerase for transcription, and identifying these promoter regions determines we were able to find the entire gene sequence of col2a1b. Due to their functional role promoter elements tend to not only be in the same location from the transcriptional start site, but also tend to show little variation in their consensus sequences (Haberle and Stark, 2018). They show little variation in their sequences due to the impact polymorphisms would have on transcription if they were present in the core promoters (Haberle and Stark, 2018). So, to find these elements, we knew to look at bases -32 to -37 upstream for BRE, bases -26 to -31 upstream for the TATA box, from bases -2 upstream to +4 downstream for INR, and then from bases +28 to +32 downstream for the DPE. Now that I knew where the transcriptional start site was located due to finding these core promoter elements, I could start looking in larger regions to look for evolutionarily conserved regions (ECR) in the upstream and downstream regions from the transcriptional start site.
